# Evaluation of an Italian Population-Based Programme for Risk Assessment and Genetic Counselling and Testing for BRCA1/2-Related Hereditary Breast and Ovarian Cancer after 10 Years of Operation: An Observational Study Protocol

**DOI:** 10.3390/mps7040063

**Published:** 2024-08-13

**Authors:** Stefano Ferretti, Priscilla Sassoli de Bianchi, Debora Canuti, Cinzia Campari, Laura Cortesi, Valentina Arcangeli, Elena Barbieri, Cecilia D’Aloia, Rita Danesi, Pierandrea De Iaco, Margherita De Lillo, Laura Lombardo, Gabriella Moretti, Antonino Musolino, Dante Palli, Caterina Palmonari, Mila Ravegnani, Alfredo Tafà, Alessandra Tononi, Daniela Turchetti, Claudio Zamagni, Valentina Zampiga, Lauro Bucchi, the HBOC Study Group

**Affiliations:** 1 Department of Morphology, Surgery and Experimental Medicine, University of Ferrara, 44121 Ferrara, Italy; stefano.ferretti@unife.it; 2 Local Health Authority, 44121 Ferrara, Italy; 3 Department of Health, Emilia-Romagna Region, 40127 Bologna, Italy; priscilla.sassoli@regione.emilia-romagna.it (P.S.d.B.); debora.canuti@regione.emilia-romagna.it (D.C.); 4 Azienda USL, IRCCS di Reggio Emilia, 42123 Reggio Emilia, Italy; cinzia.campari@ausl.re.it (C.C.); gabriella.moretti@ausl.re.it (G.M.); 5 Struttura di Genetica Oncologica, Dipartimento di Oncologia ed Ematologia, AOU Policlinico di Modena, 41125 Modena, Italy; 6 Emilia-Romagna Cancer Registry, Romagna Unit, IRCCS Istituto Romagnolo per lo Studio dei Tumori (IRST) Dino Amadori, Meldola, 47014 Forlì, Italy; valentina.arcangeli@irst.emr.it (V.A.); rita.danesi@irst.emr.it (R.D.); mila.ravegnani@irst.emr.it (M.R.); 7 Biosciences Laboratory, IRCCS Istituto Romagnolo per lo Studio dei Tumori (IRST) Dino Amadori, 47014 Meldola, Italy; valentina.zampiga@irst.emr.it; 8 Struttura di Oncologia, Dipartimento di Oncologia ed Ematologia, AOU Policlinico di Modena, 41125 Modena, Italy; barbieri.elena@aou.mo.it; 9 Section of Radiology and Breast Unit, University Hospital of Parma, 43126 Parma, Italy; cdaloia@ao.pr.it; 10 Division of Oncologic Gynecology, IRCCS Azienda Ospedaliero-Universitaria di Bologna, 40138 Bologna, Italy; pierandrea.deiaco@unibo.it; 11 Screening and Spoke Centre, 40026 Imola, Italy; m.delillo@ausl.imola.bo.it; 12 U.O. Medicina Oncologica, 41012 Carpi, Italy; l.lombardo@ausl.mo.it; 13 Department of Medicine and Surgery, University Hospital of Parma, 43126 Parma, Italy; 14 Medical Oncology, Breast Unit and Cancer Genetics Service, University Hospital of Parma, 43126 Parma, Italy; 15 UOC Chirurgia Generale a Indirizzo Senologico and Breast Unit, 29121 Piacenza, Italy; d.palli@ausl.pc.it; 16 Cancer Screening Centre and Spoke Centre, AUSL Ferrara, 44121 Ferrara, Italy; c.palmonari@ausl.fe.it; 17 UOC Senologia, Ospedale Bellaria, AUSL Bologna, 40139 Bologna, Italy; tafa.alfredo@ausl.bologna.it; 18 Unità Operativa di Prevenzione Oncologica, Ospedale Infermi, 47923 Rimini, Italy; alessandra.tononi@auslromagna.it; 19 Department of Medical and Surgical Sciences (DIMEC), University of Bologna, 40138 Bologna, Italy; daniela.turchetti@aosp.bo.it; 20 Medical Genetics Unit, IRCCS Azienda Ospedaliero-Universitaria di Bologna, 40138 Bologna, Italy; 21 Medical Oncology Unit, IRCCS Azienda Ospedaliero-Universitaria di Bologna, 40138 Bologna, Italy

**Keywords:** BRCA1/2, risk assessment, genetic counselling, genetic testing

## Abstract

Hereditary breast/ovarian cancer (HBOC) syndrome is caused by the inheritance of monoallelic germline BRCA1/2 gene mutations. If BRCA1/2 mutation carriers are identified before the disease develops, effective actions against HBOC can be taken, including intensive screening, risk-reducing mastectomy and salpingo-oophorectomy, and risk-reducing medications. The Italian National Prevention Plan mandates the creation of regional BRCA genetic testing programmes. So far, however, only informal data have been reported on their implementation. We have designed a study aimed at evaluating the results of a population-based programme for risk assessment and genetic counselling and testing for BRCA1/2-related HBOC that is underway in the Emilia-Romagna region (northern Italy). The programme—which is entirely free—includes basic screening with an estimate of the likelihood of carrying a BRCA1/2 mutation using a familial risk assessment tool, a closer examination of women with suspected risk increase, an assessment of the need for further genetic counselling and, if needed, genetic testing and risk-reducing interventions. In this paper, the design of the programme and the protocol of the study are presented. The study has an observational, historical cohort design. Eligible are the women found to be at an increased risk of HBOC (profile 3 women). The main objectives are (i) to determine the precision of the programme in measuring the level of risk of HBOC for profile 3 women; (ii) to determine the characteristics of profile 3 women and their association with the risk management strategy chosen; (iii) to compare the age at onset, histologic type, tumour stage, molecular subtype, and prognosis of breast/ovarian cancers observed in the cohort of profile 3 women with the features of sporadic cancers observed in the general female population; (iv) to determine the level and the determinants of adherence to recommendations; and (v) to determine the appropriateness and timing of risk-reducing surgery and medications. Investigating the quality and results of the programme is necessary because the best practices in risk assessment and genetic counselling and testing for BRCA1/2-related cancer and the challenges they encounter should be identified and shared. The study has the potential to provide sound empirical evidence for the factors affecting the effectiveness of this type of service.

## 1. Introduction

### 1.1. BRCA1/2-Related Hereditary Breast and Ovarian Cancer Risk

The breast cancer 1 and 2 (BRCA1/2) genes have a major role in the homologous recombination repair of DNA double-strand breaks occurring during the S phase [1]. Nuclear-localised BRCA proteins protect chromosome integrity through different mechanisms that take part in the assembly and activity of the macromolecular complexes that mediate DNA repair [2]. The loss of these tumour suppressive functions due to biallelic BRCA gene inactivation causes genomic instability and carcinogenesis. The inheritance of monoallelic germline BRCA1/2 mutations predisposes carriers, with a high penetrance, to several types of epithelial cancer [2].

The familial tendency to develop these cancers is referred to as hereditary breast/ovarian cancer (HBOC) syndrome [1]. This condition is characterised by the early onset of breast cancer (BC) and/or ovarian cancer (OC), bilateral cancers, multiple primary cancers and multiple family members with BC and/or OC and other more rare malignancies. Also, there is a well-documented relationship between germline BRCA1/2 mutation carrier status and triple-negative BC (TNBC) [3,4,5].

The cumulative risk by 70 years is 65% for BC and 39% for OC in BRCA1 mutation carriers and 45% and 11%, respectively, in BRCA2 mutation carriers [6]. The prevalence of BRCA1/2 mutations may be estimated, respectively, at 7.8% and 5.7% of total BC incidence and 13.5% and 6.6% of total OC incidence [7].

### 1.2. BRCA1/2-Related Cancer Control

If BRCA1/2 mutation carriers are identified before the disease develops, effective actions against HBOC can be implemented, which include earlier and more frequent, or intensive, screening and surgical and medical risk-reducing interventions. 

To accomplish the task of prompt identification of BRCA1/2 mutation carriers, at least four main types of BRCA testing models have been explored: (i) a population-based genetic screening of individuals without cancer; (ii) family history-based genetic screening, which involves testing individuals without cancer but with a family history suggestive of BRCA1/2 mutation; (iii) familial mutation-based genetic screening, equivalent to testing individuals free of cancer but with a known familial BRCA1/2 mutation; and (iv) cancer-based genetic screening, targeted at individuals with BRCA-related cancer [8].

### 1.3. Risk Assessment and Genetic Counselling and Testing for BRCA1/2-Related Cancer

The first of the above models is attracting increasing attention [9,10,11,12,13,14] because it allows one to identify a larger number of unaffected BRCA1/2 mutation carriers [14] and to overcome disparities [15]. However, most expert panels recommend, with limited differences, that genetic screening for BRCA1/2 mutations be based on an evaluation of personal and family history, with a risk assessment and genetic counselling if appropriate [16,17,18,19,20,21,22,23]. According to most guidelines, this process should have quality requirements. Genetic counselling for BRCA1/2 mutation testing should be performed by trained health professionals. Testing for BRCA1/2 mutations should be performed when an individual’s personal or family history suggests an inherited cancer susceptibility and when the results of the testing are expected to have an impact on the decision making. Assistance by a health professional trained in genetic counselling and testing is needed [22,24].

### 1.4. Risk Assessment and Genetic Counselling and Testing for BRCA1/2-Related Cancer and the Creation of Breast Centres

In many medical areas, the importance of providing coordinated care on a multidisciplinary basis and with a patient-centred approach is well recognised [25,26], especially when the care pathway involves multiple complex procedures. In clinical settings of this type, it is also needed to set up an infrastructure for coordination and communication in order to ensure the provision of quality care throughout the entire pathway [27].

The process of risk assessment and genetic counselling and testing for BRCA1/2-related cancer is undoubtedly of extreme complexity both in technical terms and from the perspective of the variety of actors potentially involved [22,24]. This complexity poses obvious problems of governance. Consequently, the implementation of these activities is necessarily interrelated with the creation of specialist breast centres (also referred to as breast units). A breast centre is defined as a place, or a network of places, that provides all breast care services on a multidisciplinary basis to a defined population including, in particular, genetics and prevention, the treatment of primary tumour, the care of advanced disease, supportive and palliative care, survivorship care and psychosocial support [28]. 

In the 1990s, evidence was found that patients cared for in breast centres have better outcomes [29]. In 2003 and 2006, two resolutions of the European Parliament set the deadline of 2016 for the creation of breast centres [30,31]. According to recent data, however, this deadline has been missed in many European countries [32,33]. This raises concerns as to the proper implementation of risk assessment and genetic counselling and testing for BRCA1/2-related cancer, especially with respect to between-service coordination.

### 1.5. Current Levels of Multidisciplinary Provision of Programmes for Risk Assessment and Genetic Counselling and Testing for BRCA1/2-Related Cancer

The above concerns are corroborated by the fact that, even in publicly funded healthcare systems, there are virtually no data on the prevalence of BRCA1/2-related cancer control measures at the population level (except those collected with surveys of professionals) [34,35,36] nor studies covering BRCA1/2 testing activities in their entirety. By definition, single-institution studies report only one stage of the process [37,38,39,40,41].

A comparable limitation applies to many studies focusing on BRCA1/2 mutation carriers alone, in particular those dealing with single selected issues like, for example, the effect of salpingo-oophorectomy on BC risk [42], the psychosocial well-being after risk-reducing surgery [43], and the results of magnetic resonance imaging and mammography screening [44]. This paucity of comprehensive data leaves unanswered many key questions amongst policymakers, clinicians, and women on the conduct and actual impact of programmes. 

### 1.6. Italian Recommendations and Public Health Policies on BRCA1/2 Testing

In Italy, 14 scientific societies have released a joint position paper on the implementation of preventive and predictive BRCA testing for BC, OC, pancreatic cancer and prostate cancer [23]. In agreement with national [45,46] as well as international guidelines [16,17,18,19,20,21,22], the paper states that the eligibility for BRCA1/2 mutation testing is based on personal and family history, taking into account the number of affected relatives, the type of neoplasms, the presence of multiple primary tumours, the age at diagnosis, the sex, and the immunohistochemical and molecular characteristics of tumours.

Interventions for risk assessment and genetic counselling and testing for BRCA1/2-related cancer are now part of public health strategies. The 2014–2018 Italian National Prevention Plan mandated the creation of BRCA genetic testing programmes at the regional level. The new Italian National Prevention Plan has confirmed these objectives [47]. Several regional administrations have passed local laws and regulations to implement the BRCA1/2 testing services. In fact, only informal data have been reported on the prevalence and the results of these public health activities.

All of the above considerations provided the rationale for an observational study aimed at evaluating the results of a population-based programme for risk assessment and genetic counselling and testing for BRCA1/2-related HBOC that is underway in the Emilia-Romagna region, a large administrative region of northern Italy. In the next sections of this article, we will describe, first, the overall design of the programme and the procedures being used and, second, the evaluative study protocol.

## 2. Methods

### 2.1. Programme Development

The programme was developed in 2010 by a multidisciplinary workgroup appointed by the Emilia-Romagna Regional Administration and was approved in 2011 (resolution no. 220/2011) [48]. It was launched in 2012 as a part of a wider reorganisation of BC prevention services (resolutions no. 1035/2009 and no. 1414/2012) [49,50]. In that year, the resident female population was 2,295,039. A second edition of the protocol was approved in 2016 [51]. 

### 2.2. Overall Programme Design

The programme is divided into three levels: first, a basic screening with an estimate of the likelihood of carrying a BRCA1/2 mutation using a familial risk assessment tool, which is offered to all women; then, a closer examination of women with a suspected increased risk in order to assess the need for further genetic counselling; and finally, the phase of genetic counselling with, if any, genetic testing and risk-reducing interventions. The participation in the programme is entirely free. 

For the second and third level of the programme, a hub-and-spoke organisation design was adopted. This was expected to promote the exchange of information and the standardisation of referral guidelines [52]. The programme arranges service delivery assets into a network made of four hub centres (anchor referral centres for genetic counselling and testing), complemented by 13 secondary spoke centres. The latter are in charge of investigating asymptomatic women referred from the first level of the programme and of performing a basic estimate of their likelihood of carrying a BRCA1/2 mutation by means of the Tyrer–Cuzick familial risk assessment tool [53]. The health institutions involved in the programme and their characteristics are shown in Table 1.

### 2.3. Procedures for Risk Assessment

**Figure 1:**
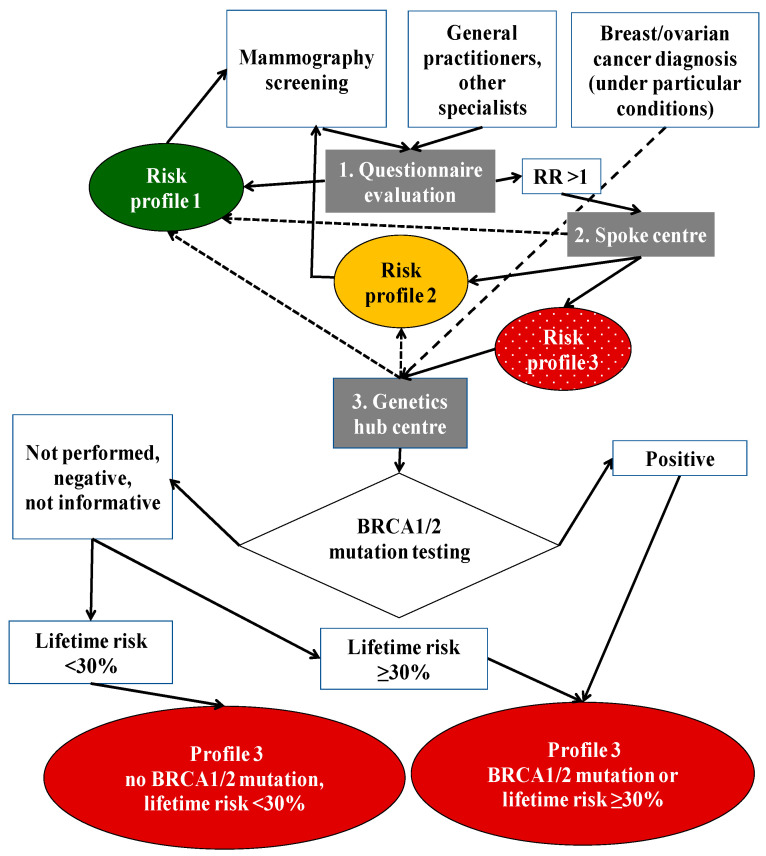
Technical scheme depicting the flow chart of the programme for risk assessment and genetic counselling and testing for BRCA1/2-related hereditary breast and ovarian cancer (Emilia-Romagna region, Italy). Profiles indicate estimates of the relative risk of developing cancer. Profile 1, women at average risk; profile 2, women at moderately increased risk; profile 3, women at high risk.

A whole flow chart of the programme is depicted in Figure 1. At all three levels of the intervention, the procedures are substantially in line with the national and international guidelines mentioned above [16,17,18,19,20,21,22,23]. The procedures for the risk assessment include the following.

- The first step is to fill out a questionnaire about the occurrence of cancer in women’s relatives (Table 2). The validation of this tool is described elsewhere [54,55].
- The questionnaire is either (i) administered periodically to women by general practitioners and breast specialists of the regional healthcare system or (ii) administered to participants in the regional breast screening programme. This is targeted at women aged 45–74 years who are invited for a 2-view digital mammography annually (at age 45–49 years) or every two years [56,57,58,59,60].
- The questionnaire assigns a score from 0 to 2 for each risk condition. Women with a final questionnaire score < 2 without a significant family history are classified as ‘not at increased risk’ (profile 1). No other action to evaluate their personal risk is taken until the next questionnaire compilation.
- The result of the questionnaire and the consequent recommendations are communicated to the women by the general practitioners or the specialists who propose it. The screening centres report the result and the recommendations in a letter communicating the result of mammography. Currently, direct contact by telephone is being activated by several spoke centres.

**Table 2:** The familial risk assessment questionnaire being used to obtain a basic estimate of a woman’s likelihood of carrying a BRCA1/2 gene mutation in the programme for risk assessment and genetic counselling and testing for BRCA1/2-related hereditary breast and ovarian cancer (Emilia-Romagna region, Italy).

Relative	Breast Cancer by Age at Diagnosis					Ovarian Cancer
	<40 Years	40–49 Years		50–59 Years	≥60 Years	Any Age
		Bilateral *	Monolateral			
Woman herself	2	2	1	1	0	2
Mother	2	2	1	1	0	1
Sister 1	2	2	1	1	0	1
Sister 2	2	2	1	1	0	1
Daughter 1	2	2	1	1	0	1
Daughter 2	2	2	1	1	0	1
Grandma ^a^	2	2	1	1	0	1
Aunt 1 ^a^	2	2	1	1	0	1
Aunt 2 ^b^	2	2	1	1	0	1
Grandma ^b^	2	1	1	0	0	1
Aunt 1 ^b^	2	1	1	0	0	1
Aunt 2 ^b^	2	1	1	0	0	1
Relative, male #	2	2	2	2	2	-
Cousin ^c^	1	0	0	0	0	1
Nephew	1	1	1	0	0	1

* First. ^a^ Paternal. ^b^ Maternal. ^c^ Father’s brother (on the paternal side, breast and ovarian cancers involve more ‘distant’ relatives than on the maternal side). # Reporting any male relative prompts referral to the hub centre. The risk score, calculated with the Cuzick–Tyrer test [51], is the ratio between the personal lifetime risk and the general population lifetime risk.

- Women with a score ≥ 2 are invited to attend the local spoke centre, unless they meet specific criteria that warrant a direct access to a hub centre. Details are shown in Table 3. If direct-access conditions are not met, the spoke centre collects a more detailed family history on BC and OC and estimates the woman’s relative risk (RR) of developing cancer, defined as the ratio between the personal lifetime risk and the general population lifetime risk, by means of the Cuzick–Tyrer test [51]. Following these steps, the spoke centre can reassign a profile 1 category of risk (average risk population) or assign profile 2 (RR = 2) or profile 3 categories of risk (high risk, RR ≥ 3). As far as profile 3 is concerned, women are invited to undergo genetic counselling at the hub genetic centre.

### 2.4. Procedures for Genetic Counselling and Testing

The procedures for genetic counselling and testing include the following:

- The hub centre performs a more in-depth evaluation of the anamnestic data, focusing on early-onset BC, BC and OC (in the patient and relatives), male BC, basal TNBC before the age of 60 years, non-mucinous and non-borderline OC, ≥2 familial BC cases with a first-degree relationship and age at diagnosis ≤40 years, and bilateral BC.
- The hub centre establishes the likelihood of germline BRCA1/2 mutation using a repeated Tyrer–Cuzick test or the BRCAPRO test [61]. The BRCAPRO probabilistic model is designed for individuals with an important personal/family history of BC and OC. The variables that the BRCAPRO model, via the reconstruction of the pedigree, allows one to incorporate refer to the first- and second-degree relatives and include gender, age, health status (healthy vs. affected by BC and OC) and result of the BRCA test if already performed.
- If well-defined risk conditions are identified (Table 4), a genetic test for BRCA1/2 germline mutations is performed. The test includes the next-generation sequencing (NGS) of the entire coding sequence and the multiple ligation polymerase analysis (MLPA) for the rearrangement of BRCA1/2 genes. The protocol considers ‘pathogenetic’ all mutations (nonsense, insertions, and deletions) which terminate prematurely the protein, specific missense mutations and ‘harmful’ mutations in non-coding regions and abnormal RNA processing. Missense and non-coding region mutations are considered ‘unclassified variants’ since the meaning of these mutations is still undetermined. If the BRCA1/2 sequences are equal to the reference normal sequences, the case is classified as unaltered. When no pathogenetic mutations are found (ENIGMA classes 1–3), the final risk classification is based on the lifetime risk (<30%, ≥30%) obtained with the Tyrer–Cuzick model. Overall, the test can be ‘positive’ (germline BRCA1/2 mutation is present); ‘true negative’ (woman is not a carrier of BRCA1/2 germline mutation already identified in her family); ‘not informative’ (the genetic test does not find any mutation in the woman or in her relatives); and ‘not conclusive’ (unknown BRCA1/2 variants found). Four final risk profiles are identified:
- Profile 1, RR = 1 (average risk);
- Profile 2 (RR = 2: moderate risk);
- Profile 3 (RR ≥ 3: high risk without BRCA1/2 mutation, lifetime risk <30%);
- Profile 3 (RR ≥ 3: high risk with BRCA1/2 mutation or lifetime risk ≥30%).

### 2.5. Risk-Reducing Interventions

#### 2.5.1. General Considerations

The programme offers risk-reducing strategies that are tailored to the woman’s profile and vary from active surveillance to systemic chemoprevention and, in women meeting stringent eligibility criteria, risk-reduction surgery. These measures are put in place to detect cancer earlier or to prevent it from developing. Great emphasis is given to psychological assistance, which is based on a non-directive approach, with the primary aims to strengthen autonomous decision making and to encourage familial resiliency. Risk profile 3 women also receive healthy lifestyle support.

#### 2.5.2. Early Detection and Screening Protocols

Profile 1–2 women are only invited to attend the regional mammography screening programme. For profile 2 women, however, this invitation is brought forward to the age of 40 years instead of 45 (Table 5). No surveillance is planned for OC. For profile 3 women, detailed schedules are established, taking age, lifetime risk and BRCA1/2 mutations into account. With respect to BC, several levels of screening intensity are provided. In the protocols of 2011 and 2016, the early detection of BC is explicitly referred to as the objective of intensive screening [48,51]. The rationale is to offer these women, who are exposed to the risk of more aggressive BC [3,4,5], a timeliness of diagnosis and a survival probability non-inferior to women diagnosed with sporadic disease, who are targeted by the standard mammography screening protocol or routine clinical surveillance (depending on age).

For OC, despite the lack of clear scientific evidence, bimanual gynaecologic examination, pelvic transvaginal ultrasound and blood Ca125 dosage every 6 months are performed.

#### 2.5.3. Chemoprevention

At the beginning of the programme, as well as after the protocol update of 2016, the chemoprevention offered to women included tamoxifen [62,63,64,65] and raloxifen [66,67] (Table 6), the latter being characterised by less adverse side effects. At the time of protocol writing, aromatase inhibitors showed initial evidence of efficacy in high-risk women, but not sufficient for its recommendation in daily practice. For OC prevention, especially at the premenopausal age, oral contraceptives have shown a protective role [68]. The low-dose use of contraceptives seems not to represent a risk for the breast [69]. Hormone replacement therapy may be indicated, with the same criteria as in the general population, in women with BRCA1/2 mutations [70,71,72]. In women with previous BC, no hormone replacement therapy should be used. With respect to PARP inhibitors, evidence of their effectiveness was considered insufficient in 2016. These agents will be reconsidered in the next protocol update.

#### 2.5.4. Risk-Reducing Surgery

As an alternative to the targeted screening protocol, profile 3 women (with or without BRCA1/2 mutation) are offered bilateral/contralateral mastectomy with complete reconstruction and a salpingo-oophorectomy. The eligibility criteria include (i) a genetic counselling profile, (ii) multidisciplinary consulting (geneticist, oncologist, radiologist, general or breast surgeon, plastic surgeon, and gynaecologist), and (iii) psychological counselling before (5 to 6 visits) and after surgery. Women younger than 50 years can choose a salpingectomy followed by an ovariectomy at the age of 50 years.

#### 2.5.5. Psychological Assistance

The active listening of patients and a non-directive approach are established with the aim to strengthen their decision-making autonomy, to encourage familial resiliency, to increase self-awareness about follow-up procedures, to support the psychological adaptation to their new condition and to help the familial communication.

#### 2.5.6. Lifestyle Support

Risk profile 3 women are offered a dedicated healthy lifestyle support programme (Table 7). 

### 2.6. Monitoring and Evaluation

The original protocol of the programme did not include guidelines for a formal and comprehensive evaluation of the related activities, which was deferred to a later time. So far, only preliminary descriptive data have been published [73].

### 2.7. Study Protocol

#### 2.7.1. Overall Design

The staff of the hub and spoke centres and the Emilia-Romagna Cancer Registry will conduct an observational, multiscope, historical cohort study aimed at obtaining a comprehensive evaluation of the results of the programme. All levels of the process will be included, namely the following: risk assessment, genetic counselling, genetic testing and risk-reducing interventions. This broad-sweeping approach will provide a general perspective on the process. The evaluation includes impact endpoints, which aim to demonstrate that the intervention has had its intended effects using a quantitative approach, as well as process endpoints, aiming to assess whether the intervention has been delivered as originally planned using a qualitative approach.

#### 2.7.2. Objectives

The specific objectives to be pursued are as follows:

1. To determine the precision of the programme in measuring the risk profile for BC and OC through the evaluation of the cumulative incidence of the two diseases relative to the age-comparable general female population of the Emilia-Romagna region after the start of the programme.
2. To determine the following:
3. The distribution of profile 2 women according to their characteristics at entry;
4. The distribution of profile 3 women according to their characteristics at entry;
5. The independent association of the characteristics of profile 3 women at entry with the risk management strategy chosen.
6. To compare (i) the age at onset, (ii) the histologic type, (iii) the TNM stage of disease, (iv) the molecular features and (v) other disease-specific characteristics of BCs and OCs diagnosed in profile 3 women with the features of sporadic incident diseases registered in the age-comparable general female population of the Emilia-Romagna region after the start of the programme.
7. To compare the 3- and 5-year net survival of profile 3 women diagnosed with BC and OC with the survival of women diagnosed with sporadic incident diseases in the age-comparable general female population of the Emilia-Romagna region after the start of the programme.
8. To assess the following:
9. The observed timing and exam composition of the surveillance protocol among risk profile 2 and 3 women who chose it;
10. The level of adherence of risk profile 2 and 3 women to the surveillance protocol;
11. The association of characteristics of risk profile 2 and 3 women with the level of adherence to the surveillance protocol.
12. To assess the time to, and the independent determinants of, risk-reducing surgery among women who first chose the strategy of intensive surveillance and risk-reducing chemoprevention. Secondary research projects are not addressed here.

#### 2.7.3. Eligibility Criteria

Women with (1) residence in the Emilia-Romagna region, (2) no previous prophylactic mastectomy, and (3) a final risk profile ≥2 are eligible for entry in the cohort.

#### 2.7.4. Data Collection and Management

Data collection and management will comply with the Italian data protection regulation. Information on women will be drawn from a variety of clinical data sources at the hub and spoke centres. Information will be recorded using a standard pro-forma datasheet. The datasheets will be submitted to the study coordinating centre as .xls files or tab-delimited text files using an existing regional network. The definition and the format of variables are available from the corresponding author upon request. 

On receipt, each dataset will be incorporated into the statistical package STATA version 15.1 (Stata Corporation, College Station, TX, USA). The centre of origin will receive direct feedback and queries for missing, erroneous or improbable data items. The working dataset will be anonymised as early as possible. Electronic data will be maintained on servers that incorporate security protections. Only the research team will have access to the study data. No published material will contain subject-identifiable information. 

To identify follow-up events, the records of women in the cohort will be linked to a regional outpatient care database and two regional drug provision databases (outpatient and inpatient provision). The regional outpatient care database (Italian: *Assistenza Specialistica Ambulatoriale* or ASA) includes individual records of services delivered to non-admitted, non-emergency patients in outpatient clinics of the National Health Service [74]. The original names of the two Regional drug provision databases are *Assistenza Farmaceutica Territoriale* or AFT and *Farmaci a Erogazione Diretta/Distribuzione Diretta Farmaci* or FED, respectively. To identify incident BC and OC cases, the records of women in the cohort will be linked to the certified Emilia-Romagna Cancer Registry [75].

#### 2.7.5. Data Analysis Plan

- Objective 1: Cumulative age-standardised (2013 European standard population) BC and OC incidence rates will be calculated. Incidence comparisons of risk profile 2 women and risk profile 3 women with the general population will be based on the incidence rate ratio (IRR) with 95% confidence interval (CI). The IRRs will be estimated with Poisson regression analysis controlling for the 5-year age group.
- Objective 2 (2a and 2b): The characteristics of risk profile 2 women and risk profile 3 women at entry will be described using means, medians, standard deviations and interquartile ranges for continuous variables, and absolute and relative frequencies for categorical variables. (2c) The multivariate analysis of the characteristics of risk profile 3 women associated with the risk management strategy chosen will be conducted using a multinomial regression model.
- Objective 3: Comparisons for age at onset, histologic type, TNM stage, molecular features and other characteristics between BCs and (as a separate population) OCs diagnosed in profile 3 women with the features of sporadic incident cancers registered in the age-comparable general female population of the Emilia-Romagna region after the start of the programme will be conducted using binary logistic regression and multinomial regression models. We hypothesise that each prognostic characteristic of diseases diagnosed in profile 3 women is non-inferior to sporadic incident cancers, with the lower bound of the 95% CI around the difference not extending beyond a margin (non-inferiority margin) to be defined by the public health authority, i.e., the Department of Health of the Emilia-Romagna Regional Administration.
- Objective 4: Three- and 5-year net survival will be calculated with the Pohar-Perme estimator [76] using the cohort approach [77]. The estimates will be age-standardised using the International Cancer Survival Standard (ICSS)-1 weights [78]. To adjust for background mortality, the Emilia-Romagna region lifetables stratified by year and patient age from the Italian National Statistics Institute will be used. A multivariate analysis of 5-year net survival will be conducted by calculating the relative excess risk of death. A flexible parametric survival model using restricted cubic splines will be fitted on the log cumulative excess hazard scale. Flexible parametric models for net survival will be fitted on individual-level data. A non-inferiority design will be used. We hypothesise that the survival from cancers diagnosed in profile 3 women is non-inferior to sporadic incident cancers.
- Objective 5 (5a): The observed timing, time intervals and exam composition of the surveillance protocols among risk profile 2 women and risk profile 3 women will be described using means, medians, standard deviations, interquartile ranges, and absolute and relative frequencies. (5b) With respect to the level of adherence of women to the recommendations set out in the surveillance protocols, the study will consider all exams available. Adherence to follow-up in each single year will be defined as an exam performed during that year ± 3 months. Adherence will be studied for each single type of exam and for the complete schedule as well. The study outcomes will be measured in the same individual in each period of follow-up. (5c) In order to take into account the fact that the repeated observations of each individual are correlated, the association between the patient and disease characteristics and compliance to periodic exams will be examined using a longitudinal model (repeated-measures logistic regression). A generalised estimating equation (GEE) model will be fitted, indicating binomial as the probability distribution and logit as the link function and specifying an autoregressive (lag-1) working correlation [79].
- Objective 6: The time to risk-reducing surgery among women who first chose the strategy of intensive surveillance and (as a distinct population) risk-reducing medication will be assessed using the Kaplan–Meier method and the log-rank test. The independent determinants of the time to risk-reducing surgery will be identified using Cox proportional hazard models.
- In all of the above analyses, the statistical significance will be set at the 5% level (*p*-value < 0.05). Borderline statistical significance will be defined as *p*-values between 0.05 and 0.10.

#### 2.7.6. Study Timeline and Recruitment Issues

The above six objectives represent six successive stages of data analysis which will be undertaken in sequence over an expected time span of no less than four years. During this period, the study datasets will be updated regularly. As a consequence, this study should at present be considered an ongoing study that has not completed yet the recruitment of patients. If the HBOC Study Group will decide to update one or more of the six analyses in the future, the datasets will be further extended.

#### 2.7.7. Preliminary Data

In order to plan the data management activities and to align them with the project schedule, we obtained routine service data collected by the Department of Health of the Emilia-Romagna region from the hub and spoke centres for management control purposes. We assumed the period 2012–2016 to represent the prevalence round of the programme. The questionnaire was proposed to 660,333 women, 660,040 (99.9%) of whom accepted. Of these, 18,155 (2.8%) were classified as profile ≥2 and were referred to spoke centres. The number of women actually seen at the spoke centres was 11,676 (64.3%). The number of women referred to hub centres was 5686, including 2815 women from the spoke centres and 2871 directly from the basic screening level because they met the criteria for direct access. Genetic testing was performed in 2431 (42.8%) women. Five hundred sixty-four (23.2%) of them were diagnosed with a BRCA1/2 mutation, which is equivalent to a prevalence of 0.86 per 1000 women entering the programme.

## 3. Concluding Remarks

### 3.1. Strengths of the Programme

The programme addresses the need for ensuring risk assessment and genetic counselling and testing for BRCA1/2-related HBOC for the whole population of the Emilia-Romagna region. All female residents, regardless of their health conditions, are actively invited to participate. The main objectives are to avoid unnecessary testing in women who are not at risk and to reach all of those who are at risk, including women with confirmed germline mutation and women with familial risk other than gene mutation.

### 3.2. Weaknesses

The last two Italian National Prevention Plans have mandated the development of BRCA genetic counselling and testing centres in all administrative regions [8,47]. This makes it necessary to investigate the results of the ongoing initiatives in order to identify the best practices as well as the sources of inadequacy. The study presented here has the potential to provide sound empirical evidence for the factors affecting the effectiveness of this type of service. The analysis of data from the entire period of operation of the programme will suggest improvements and corrections to the procedure including, if needed, changes in the BRCA testing model and in the definition of the target population [80,81]. 

The study protocol does not address the effects of enrolment in the programme on the quality of life of women. In fact, this key issue warrants a prospective study design, especially with respect to the long-term effects on those women found to be germline BRCA1/2 mutation carriers at an early age.

The programme started in 2012 and was partially updated in 2016. In those years, there was insufficient evidence to investigate a broader gene panel. However, this could be introduced in the near future. The chemoprevention protocol, too, should be updated to reflect state-of-the-art knowledge, with an implementation of the results from clinical studies.

## Figures and Tables

**Table 1:** Characteristics of the health institutions involved in the programme for risk assessment and genetic counselling and testing for BRCA1/2-related hereditary breast and ovarian cancer (Emilia-Romagna region, Italy).

Spoke Centre	Resident Population (January 2016)	Reference Hub Centre
Piacenza	147,696	Parma Medical Genetics Unit
Parma	229,847	
Reggio Emilia	271,881	Modena Medical Genetics Unit
Modena Carpi	360,002	
Bologna I Bologna II Imola	522,614	Bologna Genetics Unit
Ferrara	183,551	
Ravenna	202,297	Meldola (Forlì-Cesena) Medical Genetics Unit
Forlì-Cesena	203,552	
Rimini	174,470	

**Table 3:** Criteria prompting (if at least one is fulfilled) direct access to the hub centres in the programme for risk assessment and genetic counselling and testing for BRCA1/2-related hereditary breast and ovarian cancer (Emilia-Romagna region, Italy).

Personal history: a. Breast and ovarian cancer b. Ovarian, fallopian tube, peritoneal cancer (other than mucinous and borderline) c. Breast cancer at ≤36 years d. Male breast cancer e. Bilateral breast cancer at ≤50 years f. Triple-negative breast cancer at ≤60 years
2. First-degree relative: a. Same categories as above (patients alive for genetic examination)
3. Personal history or first-degree relative: a. Woman with breast cancer at <50 years and ≥1 first-degree relatives with the following: Breast cancer at <50 years Ovarian cancer at any age Bilateral breast cancer Male breast cancer b. Woman with breast cancer at >50 years and familial history of breast/ovarian cancer in ≥2 first-degree relatives (at least one first-degree relative to her) c. Woman with ovarian cancer and a first-degree relative with the following: Breast cancer at <50 years Ovarian cancer at any age Bilateral breast cancer Male breast cancer
4. BRCA1/2 or p53 germline mutation in the family
5. Cuzick–Tyrer risk (relative risk) >3 and BRCA1/2 positivity risk >5%

**Table 4:** Criteria prompting (if at least one is fulfilled) access to genetic testing in the programme for risk assessment and genetic counselling and testing for BRCA1/2-related hereditary breast and ovarian cancer (Emilia-Romagna region, Italy).

Both breast cancer (BC) and ovarian cancer (OC) Ovarian/fallopian tube cancer (other than mucinous and borderline), at any age, with/without family history, or occurrence of ≥1 case of OC Hereditary breast and ovarian cancer: families with ≥1 OC associated with ≥2 BCs, of which at least 1 diagnosed before 40 years of age, and first-degree relationships among 3 persons Suspected hereditary BC and OC (SHBOC): ≥3 first-degree relatives with BC/OC (not of a young age) or of a young age and with bilaterality (without first-degree relationship) Hereditary breast cancer (HBC): ≥3 BC (first-degree relatives), 1 of them before 40 years of age, or bilateral and first-degree relationship among them Strongly suspected familial BC/OC (SSBOC+): 1 BC and 1 OC with first-degree relationship and age ≤40 years or bilaterality Early onset BC (EOBC): BC at age ≤35 years without familial risk Male BC (MBC) BOC familial risk: 3 BC/OC (no HBOC, no SHBOC) SSBOC+: 2 cases in first-degree relatives, one of them at age ≤40 years or with bilateral cancer Ductal BC, grade 3, triple-negative subtype and age ≤60 years

**Table 5:** Surveillance protocol for profile 1–3 women in the programme for risk assessment and genetic counselling and testing for BRCA1/2-related hereditary breast and ovarian cancer (Emilia-Romagna region, Italy).

Risk Profile	Surveillance Protocol
Profile 1 (average risk)	45–74 years: ^1^ standard screening protocol
Profile 2 (moderate risk)	40–44 years: yearly MG ^1^
	45–49 years: standard screening protocol (yearly MG ^1^)
	50–74 years: standard screening protocol (biennial MG ^1^)
Profile 3 (high risk without BRCA mutation and lifetime risk <30%)	25–34 years: annual breast examination + US every 6 months
	35–49 years: annual breast examination + yearly MG + US (6 months after MG)
	50–69 years: annual breast examination + yearly MG
	70–74 years: screening protocol (biennial MG ^1^)
Profile 3 (high risk with BRCA mutation or lifetime risk ≥30%)	<25 years ^2^: breast examination + US every 6 months
	25–34 years: annual breast examination + US every 6 months + yearly MRI
	35–49 years: annual breast examination + US every 6 months + yearly MG + yearly MRI
	50–69 years: annual breast examination + yearly MG + yearly MRI + US (6 months after MG)
	70–74 years: screening protocol (biennial MG ^1^)

MG: mammography; US: ultrasound; MRI: magnetic resonance imaging. ^1^ Further imaging exams can be performed. ^2^ Only for a patient with breast cancer at <29 years of age and BRCA1/2 germline mutation.

**Table 6:** Chemoprevention protocol for profile 3 women in the programme for risk assessment and genetic counselling and testing for BRCA1/2-related hereditary breast and ovarian cancer (Emilia-Romagna region, Italy).

Risk Profile	Chemoprevention Protocol
Profile 3 without BRCA germline mutation	Tamoxifen and raloxifen in women ≥35 years with a life expectancy of at least 10 years and cancer risk >1.7% at 5 years or with lobular carcinoma in situ
	Anastrazole, letrozole, examestane: not recommended yet
Profile 3 with BRCA germline mutation	Fenretinide: menopausal women (study in progress for young carriers)
	Other drugs: insufficient evidence
Ovary BRCA germline mutation without previous BC	Low-dose oral contraceptives for those of childbearing age
Ovary BRCA germline mutation with previous BC	No hormone replacement therapy

**Table 7:** Lifestyle support guidelines for profile 3 women in the programme for risk assessment and genetic counselling and testing for BRCA1/2-related hereditary breast and ovarian cancer (Emilia-Romagna region, Italy).

#	Recommendation
1.	Maintain a normal weight
2.	Practice physical activity
3.	Have fresh vegetables and fruit
4.	Avoid alcohol consumption
5.	Avoid hypercaloric food
6.	Avoid sugar consumption (sugary drinks)
7.	Limit red meat consumption
8.	Limit salt and salted meat consumption
9.	Do not use supplements for cancer prevention

## Data Availability

The datasets generated during the current study are not publicly available due to the Ethics Committee’s restrictions but are available (in Italian) from the corresponding author upon reasonable request.
